# Impact of the Application of Gaseous Ozone on Selected Pathogens Found in Animal Shelters and Other Facilities

**DOI:** 10.3390/ani13203230

**Published:** 2023-10-17

**Authors:** Veronika Vojtkovská, Dana Lobová, Eva Voslářová, Vladimír Večerek

**Affiliations:** 1 Department of Animal Protection and Welfare and Veterinary Public Health, Faculty of Veterinary Hygiene and Ecology, University of Veterinary Sciences Brno, Palackého tř. 1946/1, 612 42 Brno, Czech Republic; voslarovae@vfu.cz (E.V.); vecerekv@vfu.cz (V.V.); 2 Department of Infectious Diseases and Microbiology, Faculty of Veterinary Medicine, University of Veterinary Sciences Brno, Palackého tř. 1946/1, 612 42 Brno, Czech Republic; lobovad@vfu.cz

**Keywords:** ozone generator, pathogen surveillance, cytopathic effect

## Abstract

**Simple Summary:**

In animal shelters and other facilities wherein a high concentration of animals is typical, proper disinfection procedures are a critical to maintain the population in good health. The aim of the study was to verify the virucidal effect of gaseous ozone produced by commercially available generators. Two ozone generators were used to produce ozone and the viabilities of four viral pathogens (feline coronavirus, canine coronavirus, feline calicivirus, feline parvovirus) were tested under experimental conditions. The results of the study confirm the hypothesis of a lower resistance of enveloped viruses to gaseous ozone.

**Abstract:**

Correctly selecting disinfection procedures is crucial in facilities housing a high number of animals as it directly affects their health. The aim of this study was to verify the virucidal effect of gaseous ozone delivered by commercially available generators under controlled experimental conditions on a selection of viral pathogens (feline coronavirus, canine coronavirus, feline calicivirus, feline parvovirus) commonly found in shelters and other facilities. Two ozone generators with outputs of 3.5 g/h and 20 g/h were used to produce ozone. Virus viability after the application of ozone was evaluated by examining for typical pathogen-specific cytopathic effects on the CRFK (Crandell–Rees Feline Kidney) cell line post-incubation. No cytopathic effect was observed in feline coronavirus after the 2-h application of ozone; in canine coronavirus, the absence of a cytopathic effect was observed after the 4-h application of ozone. The absence of a cytopathic effect in feline calicivirus was observed after the 6-h application of ozone; the viability of feline parvovirus was not impaired even by the 6-h application of ozone. The results of the study confirm lower resistance to the application of gaseous ozone in enveloped viruses.

## 1. Introduction

In animal shelters and other facilities wherein a high concentration of animals is typical, properly selected disinfection procedures are a critical aspect for maintaining the population in good health as they help prevent the spread of infectious agents. The selection of a suitable disinfectant is crucial; it should take into account the target environment, the resistance of pathogens, the method of application, the duration of effect, and price [1]. Although recommendations concerning environmental sanitation in shelters concentrate mainly on traditional methods of disinfection using disinfectants (chemicals based on quaternary ammonium compounds, sodium hypochlorite, calcium hypochlorite, and hydrogen peroxide) [2], recently, owing to the COVID-19 pandemic, the discourse surrounding sanitation procedures has also expanded to include other, less conventional methods of disinfection. These include the treatment of indoor areas and spaces using so-called ozone generators, i.e., devices that deliver gaseous ozone, which is the second strongest oxidizing agent after fluorine [3]. Ozone is characterized by its ability to react with organic molecules containing double or triple bonds and this property is the cause of its bactericidal, virucidal, and fungicidal effects [4]. The effects described are utilized, for example, in water disinfection [5], and in the food industry for the disinfection of facilities, surface preservation of food products, and surface treatment of vegetables and fruits [3]. Ozone can be also successfully used in veterinary medicine as an alternative therapy in the treatment of some infectious diseases, e.g., canine parvovirus as documented by Dos Santos et al. [6].

Ozone is an unstable gas and its rate of decay depends primarily on the temperature and humidity of the environment, which ultimately affects its effectiveness; with an increase in temperature and humidity, its half-life is reduced [7]. Ozone half-life data vary across literature in accordance with the type of medium where it is present—if dispersed in air, the half-life can be 0.5 h, but can reach as much as 25.4 h under the conditions where there is an absence of airflow, a temperature of 24 °C, and zero humidity [7]. In an aquatic environment, the literature indicates a half-life of 330 s at 25 °C. Also, pH is the factor influencing half-life in aquatic environments—the higher its value, the shorter the half-life [8].

The effect of ozone on bacteria is complex as it acts directly on several cell structures including proteins, enzymes, and unsaturated fatty acids in cell membranes [9]; in gram-negative bacteria, the primary area of effect comprises lipoproteins and lipopolysaccharides. The disruption of these structures leads to an increase in the permeability of cells and their subsequent lysis [9]. In the case of viruses, the mechanism of action of ozone lies in the disruption of the surface proteins of the capsid and membrane receptors. The action of ozone affects the structure of the virus and its infectious capacity because the receptors that the virus uses to bind to host cells are altered by oxidation. Additionally, by means of phospholipid peroxidation, ozone disrupts the membranes of enveloped viruses and creates reactive oxygen species [10,11]. Inactivation of the virus may also be caused by damage to its genetic information (DNA or RNA destruction) [12]. 

There are several methods of ozone production, three of which are currently commercially available—corona discharge, UV radiation, and electrolysis [13]. To produce ozone, generators can use air or pure oxygen from a gas tank (oxygen can also be obtained from air by membrane separation). Ozone generators designed for household use, which are commonly available on the market, usually produce ozone by applying electric discharges on the air (corona discharge). High voltages pass through the dielectric to a grounded sieve or plate, thereby dividing the oxygen molecules into individual atoms. The free atoms then bind to another oxygen molecule, forming ozone. Ozone is ordinarily a highly reactive gas of blue color (colorless in low concentrations) characterized by a distinctive odor [14]. The application of gaseous ozone seems to have a number of advantages over solid and liquid chemicals used for disinfection including the ability of gas to penetrate into all parts of the treated environment, including crevices, furniture, and fabrics [15], and the fact that it does not leave harmful residues behind [11]. The ability of gas to penetrate materials is especially useful in an environment consisting of a multitude of objects (in shelters and kennels, these could include cat climbing trees, beds, and other objects made of fabric) that are otherwise relatively demanding to clean by common methods. 

Because the potential for the use of ozone generators in veterinary and animal-keeping facilities (shelters, catteries and kennels, veterinary practices, and clinics) has not been described in detail, particularly regarding viral agents, the aim of this study was to experimentally verify the impact of ozone application on significant pathogens of viral origin and varying resistances (feline coronavirus (FCoV), canine coronavirus (CaCoV), feline calicivirus (FCV), and feline parvovirus (FPV)), the occurrence of which are common in environments with a great number of animals. These viruses were chosen as they represent pathogens significantly implicated in increasing animal morbidity and mortality. They are characterized by rapid transmission and, mainly in the case of parvovirus and calicivirus, high environmental resistance. Removal from the environment is one of the key strategies to prevent the spread of pathogens in an animal population.

## 2. Materials and Methods

Field isolates of feline coronavirus, feline parvovirus, and feline calicivirus, as well as the type strain of the canine coronavirus, were used as positive control strains. The strains were isolated on a cell line of feline origin (Crandell–Rees Feline Kidney; CRFK) (Sigma-Aldrich, Burlington, MA, USA) under standard conditions, verified by PCR analysis and subsequently sequenced. The sequences were compared with strains in the GenBank database and their positivity was confirmed.

### 2.1. Preparation of Samples

Prior to use, frozen virus samples were left in a refrigerator (4 °C) for gradual thawing. TCID50 was calculated for all pathogens (FCoV TCID50 = 10^1.5^/1 mL, CaCoV TCID50 = 10^2.1^/1 mL, FCV TCID50 = 10^2.5^/1 mL, FPV TCID50 = 10^2.1^/1 mL). Pathogen samples were pipetted onto Petri dishes (each Petri dish contained a total of 3 drops of one pathogen (triplicate) in a volume of 90 µL).

### 2.2. Experimental Procedure

**Figure 1:**
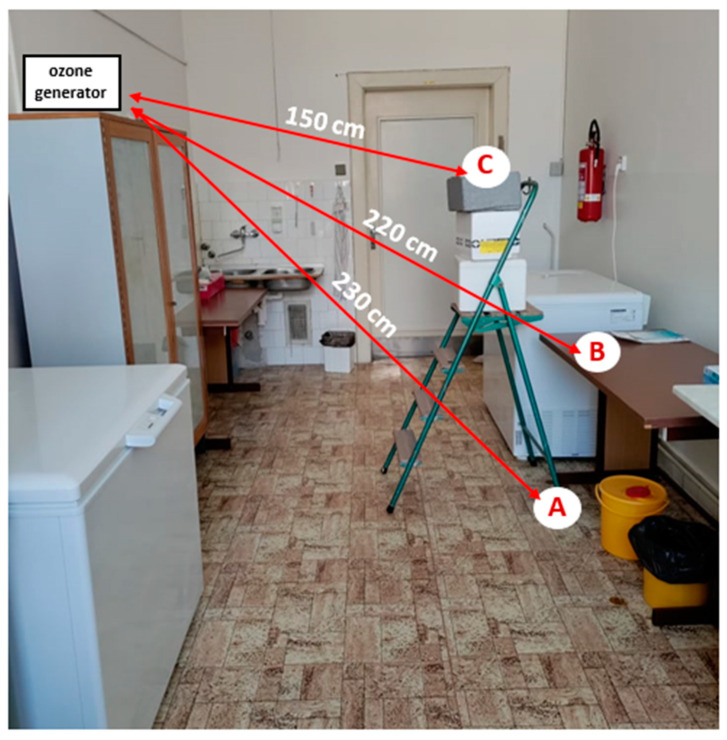
Placement of samples and devices delivering ozone in the experimental room (A: floor level; B: desk (height 77.5 cm); C: ladder with box (height 152 cm)).

Exposure of pathogens to ozone gas application was carried out in a room of an unused laboratory volume 70.3 m^3^ (2.86 m (width) × 6.70 m (length) × 3.67 m (height)) at the Department of Infectious Diseases and Microbiology, University of Veterinary Sciences Brno. The average temperature and humidity in the room during individual measurements were 25.5 °C and 55.25%, respectively. At the time of the experiment, the room was only equipped with basic work-related appliances (e.g., table, chairs, wardrobe, switched-off freezer units, storage boxes). Petri dishes containing samples of pathogens were placed in the room at 3 selected sites representing 3 different heights (A: floor level; B: desk (height 77.5 cm); C: ladder with box (height 152 cm)) to verify the impact of sample location on the effectiveness of ozone application. Two different devices (hereafter referred to as A’ and B’) were used to produce the gaseous ozone in order to verify the impact of performance on the viability of the pathogens. Only one of the devices was in operation at any given time during the experiment. The technical specifications relating to the devices used are given in Table 1. The device delivering the gaseous ozone was placed at a height of 178 cm and at a mean distance of 2 m from each sampling site (Figure 1). Both devices applied the principle of corona discharge to produce ozone from the air. Ozone generator B’ also generated ozone using short-wave ultraviolet radiation (a UV lamp was a part of device B’).

Instructions provided by the manufacturer for each device were taken into account when deciding the duration of ozone application (based on the size of the room); the duration was also adapted for specific pathogens, taking into account the results obtained over the entire duration of the study. The efficiency of two-, four-, and six-hour ozone applications was investigated. During the experiment, the door to the room was sealed with a cloth to minimize the leaking of the gas to outside the room. The concentration of gaseous ozone at different sites in the experimental room (sites A, B, C where samples were placed, output of ozone generator, window of the experimental room) was monitored during the experiment using a Triotech HR-ZE25 (Triotech, Uherské Hradiště, Czech Republic) device with a measuring range of up to 100 ppm. The single measurement of ozone concentration took place after the room was completely saturated with gas ozone and its concentration had stabilized (1 h after the ozone generator was switched on). After the ozone exposure of the samples, a person equipped with protective gear entered the room, stopped the operation of the device, and took the Petri dishes with the pathogen samples to another room to eliminate the effect of prolonged residual ozone concentration in the air on the samples. The samples remained in the post-application room for a minimum of 45 min in order to ensure the decay of any residual ozone they could potentially contain.

### 2.3. Verification of Pathogen Viability

After the samples had been left in the post-application room for at least 45 min, each sample was pipetted from the Petri dish into an Eppendorf tube containing 1500 µL of 2% DMEM medium. If the sample was in a dry state after the experimental procedure, it was removed from the Petri dish using a drop of the medium, which was applied onto the sample by pipette. The mixture of sample and medium was then filtered through a bacteriological filter (Sarstedt, Nümbrecht, Germany) into a sterile Eppendorf tube. Subsequently, the samples were inoculated under aseptic conditions into a 24-well microtitre plate containing a CRFK cell monolayer (Crandell-Rees Feline Kidney) (Sigma-Aldrich, USA). Pathogen-infected cells were cultured in a thermostat at 37 °C with a carbon dioxide concentration of 5%. The cultivation period varied depending on the specific pathogen (3 days for FCoV and CaCoV, 2 days for FCV, and 5 days for FPV). 

Positive and negative controls were cultured concurrently with the samples. Positive controls comprised pathogen samples applied to the CRFK cell line at the same dilution and amount as the sample to which the experimental procedure was applied. Control samples were left without exposure to ozone in another room. The length of time the samples were left in this room was the same as for ozone-exposed samples, plus an additional 45 min. To confirm that storage did not affect the viability, virus samples were cultured right after thawing (without exposure to ozone) concurrently with the positive control. The negative control comprised a CRFK cell line with 2% DMEM medium. After the culture period, samples exposed to ozone and positive and negative control samples were examined under an inverse microscope (Leica, Wetzlar, Germany) to verify the presence of a cytopathic effect (CPE). Cell lines were examined even after the usual period of cultivation of individual pathogens in order to detect any delay in the onset of the cytopathic effect. In cases of an unclear result (presence of visible changes in cell culture but absence of cytopathic effects typical of the given pathogen), the samples were passaged into a new culture vessel and cultured as described above. 

### 2.4. Evaluation of Cythopathic Effect Specificity by Using of PCR Analysis

Specificity of CPE for each virus was verified using of real-time PCR analysis. The procedure included RNA isolation and the transcription of RNA into cDNA using the Transcriptor First Strand cDNA Synthesis Kit (Roche, Basel, Switzerland) with random oligonucleotides for cases of FCoV, CaCoV, and FCV. In the case of FPV, DNA was extracted using the NucleoSpin extraction kit (Macherey Nagel, Nordrhein-Westfalen, Germany). Both the detection and quantification of FPV and FCV were performed using Xceed qPCR SG 1step 2 × Mix Lo -ROX (Institute of Applied Biotechnologies, Prague, Czech Republic) in accordance with the manufacturer’s instructions on a Light Cycler^®^ 480 II (Roche, Basel, Switzerland). In the cases of FCoV and CaCoV, detection by RT-nested PCR was performed using a RT-PCR kit (Qiagen, Hilden, Germany).

### 2.5. Statistical Analysis

To test the sites’ impact on the occurrence of viral cytopathic effects, data obtained from individual sites were analyzed using the statistical software Unistat 6.5 for Excel (Unistat Ltd., London, UK). Differential evaluation of the number of wells with the occurrence of a cytopathic effect within the individual sites A, B, and C was carried out by the χ^2^ (Chi-square) test. The value of *p* ≤ 0.05 was considered statistically significant.

## 3. Results

During the operation of the ozone generators, ozone concentration was monitored in various selected sites of the experimental room; the values measured for both ozone generators are given in Table 2. The highest ozone concentration was detected at the output of the ozone generator, the lowest at the level of the window of the experimental room.

The placement of the sample at a particular site (A, B, or C) did not affect the efficacy of ozone application; the difference between the number of samples in which a cytopathic effect was observed did not differ statistically significantly across sites (*p* > 0.05). The results concerning the viability of pathogens in individual experiments are presented in the following part of the chapter.

### 3.1. Verification of Viability of FCoV and CaCoV

**Figure 2:**
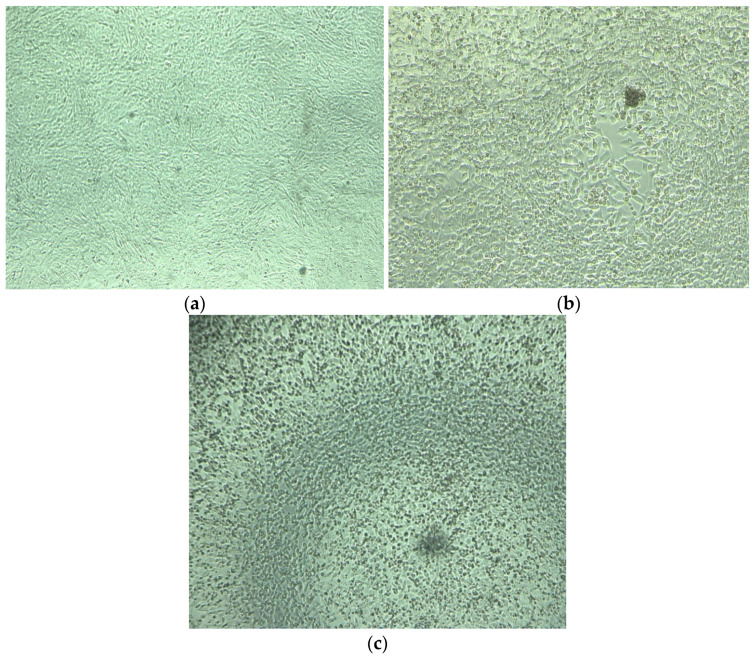
(**a**) Negative control–CRFK cells without the presence of the pathogen; (**b**) cytopathic effect of CaCoV after the 2-h exposure to ozone produced by ozone generator A’ (site B); (**c**) positive control–CRFK cells with FCoV (cytopathic effect).

The results concerning the viability of FCoV and CaCoV after the application of gaseous ozone are presented in Table 3. All samples with CPE were in accordance with the positive results obtained by PCR. Owing to the fact that the cytopathic effect was not detected in FCoV or CaCoV after 4 h of application of ozone by the ozone generator A’ (lower-power device), the experiments with these pathogens were not repeated after 6 h. For the same reason, the experiments were not repeated with the ozone generator B’ (higher-power device). Figure 2 illustrates the negative control (CRFK cells without the presence of a pathogen), cytopathic effect of CaCoV after the 2-h exposure to ozone produced by ozone generator A’, and cytopathic effect of FCoV (positive control).

### 3.2. Verification of Viability of FCV

**Figure 3:**
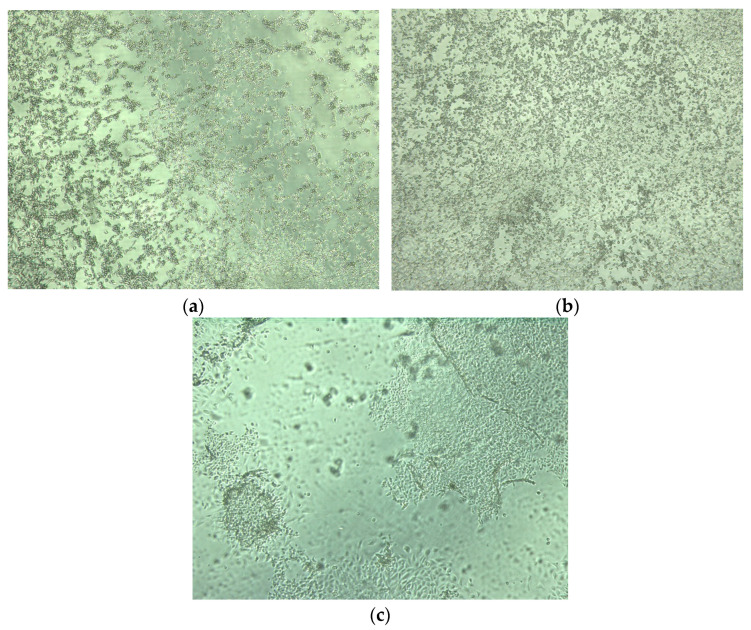
(**a**) Positive control–cytopathic effect on CRFK cells caused by FCV; (**b**) cytopathic effect of FCV on CRFK cells observed after a 2-h exposure to ozone produced by ozone generator A’ (site C); (**c**) detachment of part of CRFK cells with FCV from the bottom of the well.

The cytopathic effect of FCV was not observed on CRFK in the case of 6-h ozone exposure (ozone produced by ozone generator B’). All the samples with CPE were in accordance with the positive results obtained by PCR. All other exposures to ozone resulted in a confirmation of FCV survival as the cytopathic effect was observed (Table 4). Considering the survival of the virus after a 4-h exposure to gaseous ozone produced by ozone generator B’, the 2-h exposure was not tested. Figure 3 illustrates positive control–cytopathic effect found on CRFK cells caused by FCV, cytopathic effect of FCV found on CRFK cells observed after a 2-h exposure to ozone produced by ozone generator A’, and detachment of part of CRFK cells with FCV from the bottom of the well found at site A after a 6-h exposure to ozone produced by ozone generator A’.

### 3.3. Verification of Viability of FPV

**Figure 4:**
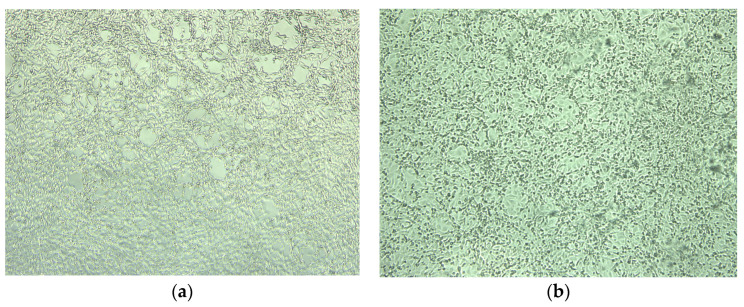
(**a**) Positive control of FPV after cultivation on the CRFK cell line (cytopathic effect); (**b**) cytopathic effect of FPV found on CRFK cells after 6 days of cultivation (6-h exposure to ozone produced by ozone generator B’ (site C)).

The exposure of FPV to gaseous ozone did not affect its viability in any of the experiments (Table 5). The cytopathic effect was observed on the cell line even after a 6-h exposure to ozone (ozone produced by ozone generator B’). All samples with CPE were in accordance with the positive results obtained by PCR. Because of the expected high resistance of the virus, exposures lasting 4 and 6 h were not carried out. Figure 4 illustrates positive control of FPV after cultivation on the CRFK cell line (cytopathic effect) and cytopathic effect of FPV found on CRFK cells after 6 days of cultivation (6-h exposure to ozone produced by ozone generator B’).

## 4. Discussion

The use of commercially available ozone generators has recently become very popular, further promoted by the COVID-19 pandemic and the related increased interest in various, less-traditional methods of disinfection. Given that some shelters (but also individual breeders and veterinary clinics) have responded to the offers of producers and distributors of ozone generators by purchasing and using them in an effort to maximize the effectiveness of disinfection, it was in the general interest to experimentally verify their effectiveness and verify the claims of manufacturers who report the elimination of a wide range of pathogens. Two commercially available ozone generators with theoretical outputs of 3.5 g/h (ozone generator A’) and 20 g/h (ozone generator B’), respectively, were used to produce ozone. The actual concentration measured, however, was significantly lower owing to ozone leakage, decay, and interactions with materials. Ozone concentration values measured during the experiment reached 0.2–0.7 ppm in the case of ozone generator A’ (at the generator output, a concentration of 12 ppm was measured), and for ozone generator B’, concentrations of 3.6–5.1 ppm were recorded (at the generator output, up to 50 ppm was measured). Although ozone is heavier than air, its concentration did not differ among sample sites located at different high levels. Additionally, there was no observed effect of sample placement on the number of cell culture wells in which a cytopathic effect of selected viral pathogens was detected after cultivation.

For testing, pathogens of viral origin (FCoV, CaCoV, FCV, and FPV) with varying levels of resistance, commonly found in shelters and other facilities with higher concentrations of cats, were used. Coronaviruses are enveloped RNA viruses that can be eliminated from the environment relatively easily using common disinfectants [16]. A virus that may be considered more resistant (characterized by a high level of resistance to many disinfectants [17]) is the feline calicivirus, a non-enveloped RNA virus able to survive in the environment for 1 month [18]. An extremely long survival time in a shelter environment [19] (potentially even longer than 1 year), resistance to high temperatures, low pH [20], and resistance to most quaternary ammonium compounds and alcohol disinfectants [21] are characteristics of FPV, a non-enveloped DNA virus that is emitted into the environment in large quantities by infected cats through saliva, urine, feces, and vomit [22].

Our findings confirm the trend presented in other studies that assume lower resistance to the application of gaseous ozone in enveloped viruses [23] probably due to the oxidation of lipids contained in the viral envelope [24]. In addition to the type of pathogen, several other factors may affect the virucidal potential of ozone concentrations, such as the temperature and humidity of the environment, and exposure time [12]. No cytopathic effect was detected in FCoV after a 2-h exposure to ozone produced by the lower-power device (ozone generator A’); in CaCoV, viability was impaired after a 4-h exposure. While lower ozone concentrations and shorter exposure times were sufficient to deactivate both types of coronaviruses, up to 6 h of exposure and higher ozone concentrations delivered by device B’ were necessary to deactivate FCV. FPV activity was not impaired even after 6 h of ozone application at a higher concentration produced by ozone generator B’. However, a delayed onset of cytopathic effect was recorded for this pathogen, which was observed in the cell line 6 days after inoculation. A similar phenomenon was observed in FCV after the 4-h exposure to ozone delivered by device B’ and in CaCoV after the 2-h exposure to ozone produced by device A’. These findings point to the effect of ozone exposure even at shorter exposure times—it is possible that exposure to ozone affected the mechanism of attack or destruction of cells; another explanation could be a scenario where the total concentration of the virus was reduced (i.e., the destruction of a portion of the viral particles and thus a decrease in virucidal capacity), manifested in the delayed onset of cytopathic effect. 

In the product description, one manufacturer of the ozone generator A’ states its effectiveness against bacteria, fungi, viruses, and odors; another manufacturer highlights mainly the elimination of fungi and odors. According to both manufacturers, generator A’ is suitable for spaces of sizes ranging from 20 to 150 m^2^; the recommended application time for the room size utilized for the purposes of this study is 6 h. The manufacturer of generator B’ declares its capability to effectively remove odors, fungi, and carcinogenic and allergenic agents in floors, walls, and ceilings; another provider from whom it is possible to rent the generator B’ for a fee also advertises its capability to eliminate viruses and bacteria, allergens, insects, and mites. This generator is primarily intended for rooms of up to 56 m^3^, but according to the manufacturer it can also be used in larger rooms if the ozone application time is extended. The recommended application time in rooms of up to 56 m^3^ is 40 min; in our study, the application time was extended to as much as 6 h. Based on the results, it can be concluded that the expected virucidal effect claimed by the manufacturers was only partial—from the pathogens subjected to testing, the only pathogen for which the cytopathic effect was not observed after ozone exposure were coronaviruses. Although the application of the ozone delivered by ozone generator B’ also led to elimination of FCV, the duration of exposure for this pathogen was 6 times longer than the manufacturers’ recommendation—if the duration specified by the manufacturer were to be followed, the application would not be effective. The evaluation of the effect of gaseous ozone on FCV viability was also the subject of the study conducted by Hudson et al. [15]; the authors confirmed the inactivation of the virus by ozone that was delivered by an ozone generator. However, in this case, the ozone concentration was 5 times higher (20–25 ppm) than the concentration developed by ozone generator B’ in our study. Short exposure times are sufficient at higher concentrations—in the given study, it was only 20 min. The lettuce samples inoculated with FCV-F9 were exposed to ozone at 5 ppm in a study by Gobeil et al. [25]. Ozonation was performed with an ozone generator for 0, 2.5, 5, 7.5, 10, and 15 min. The authors reported a 90% reduction of FCV-F9 on the surface of the iceberg lettuce leaves after 15 min of ozonation. A duration of 5 to 10 min of exposure to O_3_ at 6.25 ppm (0.9 g/h) was needed to decrease titers of FCV on lettuce and green onions in a study by Hirneisen et al. [26]. The authors attributed this reduction to the organic composition of the vegetable, as ozone reacts with the complex organic compounds in food because of the high oxidation potential [26].

Although, in our study, the application of ozone was ineffective against non-enveloped viruses when the manufacturers’ instructions were followed, the absence of a cytopathic effect observed in coronaviruses may indicate a purpose in utilizing generators at least for this type of pathogen. The elimination of FCoV from an environment with a higher concentration of cats may result in a reduction in the rate of transmission of infection between animals, which also indirectly affects the reduction in the incidence of the development of feline infectious peritonitis, which can emerge as a result of a mutation of the enteric form of the coronavirus [27]. However, it should be noted that in comparison with experimental conditions, the effectiveness of ozone application can be significantly lower under real conditions, that is, in shelters and other facilities. FCoV is excreted into the environment by animals via feces [28]; however, by coming in direct contact with feces and litter, animals can also transmit the virus to other parts of the housing facilities which usually comprises various surfaces. In this study, the effectiveness of ozone application was tested only on the plastic surface of the Petri dish; furthermore, real fecal samples were not used in testing (virus isolates contained in a liquid medium were used).

The potential use of ozone generators for the elimination of coronaviruses has been studied mainly in relation to human pathogens; many studies were spurred by the COVID-19 pandemic, which drew attention to the necessity of compliance with hygiene standards. Considering that biosafety level 3 is necessary for the handling of SARS-CoV-2, substitute, biologically safe pathogens (e.g., coronaviruses of the genus Alpha-HCoV-229E [29], influenza A virus, respiratory syncytial virus, and a number of others [30]) with a similar form, structure and function were used across the studies. Studies have come to different conclusions about the effectiveness of ozone application on these pathogens. In these studies, the ozone concentration ranged from 0.1 to 200 ppm, and the application time from 13.8 s to 320 min; high concentrations of ozone have been achieved because of the use of chambers and other small enclosed systems [30] that prevent ozone leakage. However, these experimental systems are removed from real conditions where the disinfection of larger spaces is needed. The effects of ozone exposure are questionable when objects of larger dimensions are used (experimental rooms of different sizes). The National Reference Laboratory for Disinfection and Sterilization of the National Institute of Public Health (NIPH) of the Czech Republic conducted testing to assess the effects of generator-delivered gaseous ozone on selected pathogens (*Staphylococcus aureus*, *Pseudomonas aeruginosa*, microscopic fibrous fungi *Aspergillus brasiliensis*, and model *E. coli* virus, bacteriophage φX 174) in real conditions in premises of 3 different sizes (22.5, 62, and 87.5 m^3^, respectively). Ozone concentrations ranged from 6.3 to 34 ppm and durations of application from 20 min to 4 h and 9 min. Bactericidal, fungicidal, or virucidal effects were not demonstrated in any of these experiments [31].

Repeated experiments incentivized us to set the time the samples spent in the post-application room to a minimum of 45 min. In case of absence or shortening of this time, we observed massive changes on the cell culture manifested by the detachment and the destruction of the cell monolayer. Although all samples were left in the post-incubation room for at least 45 min, detachment was observed in some samples even after this time (Figure 3c). We assume that the destruction of cells was probably caused by the residual ozone; i.e., its free radicals (hydrogen peroxide and hydroxyl), which emerged as part of the decay of ozone in the liquid (in the DMEM medium, which was used to sustain CRFK cells). Another explanation for the destruction could be the presence and effect of nitrogen compounds on cells in the liquid medium. Generators that produce ozone from the air additionally create intermediate byproducts of air ionization—diatomic molecules O_2_, N_2_, and their combinations (NO, NO_2_) are formed from ionized oxygen and nitrogen molecules. The presence of NO_2_ in the experimental room during the ozone application period was confirmed using the BW GasAlert Micro 5 measurement device. The concentrations of NO_2_ at sites A, B, C, near the door and window of the experimental room ranged between 0.5–1 ppm, while at the output of the ozone generator, the concentration was greater than 20 ppm. Air input into the generator should be at its driest to maintain the device’s durability; humid air from which nitrogen oxides are produced damages the generator. The input gas also influences the device’s efficiency; 1–3% of ozone is formed when air is used, while, when using pure oxygen, ozone formation increases to 16% [13].

The issue of using ozone generators lies not only in their effectiveness but also in their safety. In order to inactivate FPV, whose cytopathic effect was detected on the cell line despite the 6-h exposure to ozone, it would likely be necessary to develop higher ozone concentrations, which, using commercially available generators, is problematic in larger rooms (where windows and doors may additionally cause gas leaks). Inactivation of more resistant viruses could theoretically be accomplished by prolonging the application of ozone; however, exposure of as long as 6 h is already impractical in shelters and other facilities with higher animal density. Furthermore, ozone has strong irritation effects on the conjunctiva and respiratory tract, and at higher concentrations, it can cause difficulty breathing and an inflammatory reaction of the respiratory mucosa. Ozone concentrations of about 200 μg/m^3^ (0.1 ppm) may cause short-term acute effects in humans, which manifest as eye irritation. Exposure to 0.5 ppm lasting one hour can have significant harmful effects [31]. According to the WHO, the 8-h mean ozone concentration should not exceed 100 μg/m^3^ [32]. In accordance with the legislation of the Czech Republic, human ozone limits are set by Government Regulation No. 361/2007 determining conditions of occupational health protection [33]; the permissible exposure limit is 0.1 mg/m^3^ (8-h mean), the highest permissible concentration being 0.2 mg/m^3^ (mean concentration of the substance measured for up to 15 min). Ozone limits were also set for various animal species—in cats, Mittler et al. [34] reported that the LC50 value in a 3-h exposure to ozone is 34.5 ppm. The manufacturers of the generators used in this study warn of possible negative effects to human and animal health; during the application, neither animals nor persons should be present in the treated area, and, after treatment, it is recommended to ensure thorough ventilation for 30 to 60 min. Although usage of ozone generators not in accordance with instructions may cause harm to animal and human health (and this is especially true for higher-power generators), the sale of ozone generators is not regulated in the Czech Republic and practically anyone can purchase one.

The need to relocate animals away from treated areas is one of the main disadvantages of using ozone generators. When it comes to generators for which the ozone output does not exceed the safety limit, allowing their operation even in the presence of animals, their efficiency is very low or virtually absent. The US EPA (United States Environmental Protection Agency) further highlights this fact in a comment on the application of ozone for the purposes of air purification; the EPA summarizes that in concentrations not exceeding public health standards, ozone has no bactericidal and virucidal effects, nor does it remove mold or other pollutants [35]. 

## 5. Conclusions

The results of our study corroborated findings of lower resistance to the application of gaseous ozone in enveloped viruses. Based on our findings, a certain level of disinfection potential can be assumed in the case of FCoV and CaCoV; however, the virucidal effect is questionable under real animal-keeping conditions (where fecal samples of various consistencies may be found on various surfaces). In order to achieve a virucidal effect, higher concentrations of ozone must be delivered against more resistant pathogens; this is difficult when using commercially available generators. Furthermore, ozone concentrations should be maintained at levels that are safe for animals and humans.

## Figures and Tables

**Table 1:** Technical specifications, provided by the manufacturers, of the devices generating ozone.

Device Label	Ozone Production	Power Supply	Input Power	Dimensions	Weight
A’	3.5 g/h	230 V	55 W	20 × 20 × 18 cm	2.2 kg
B’	20 g/h	230 V	200 W	29.5 × 17.5 × 26 cm	5.9 kg

**Table 2:** Ozone concentration in the room measured using the Triotech HR-ZE25 device.

Site of Measurement	Ozone Generator A’ (ppm)	Ozone Generator B’ (ppm)
site A	0.5–0.7	4.3–4.9
site B	0.2	4.6–5.1
site C	0.5	3.6–5
device output	11.8–12	40–50
window of the room	0.5	3

**Table 3:** Evaluation of the viability of FCoV and CaCoV after exposure to gaseous ozone (CPE+/−: presence/absence of cytopathic effect).

Pathogen	Duration of Exposure	Ozone Generator	Result		
			Site A (CPE+/−; Number of Wells with CPE)	Site B (CPE+/−; Number of Wells with CPE)	Site C (CPE+/−; Number of Wells with CPE)
FCoV	2 h	A’	−	−	−
FCoV	4 h	A’	− ^a^	− ^a^	− ^a^
CaCoV	2 h	A’	+ (3/3) *^,b^	+ (3/3) *	+ (3/3) *
CaCoV	4 h	A’	−	−	−

* Delayed onset of cytopathic effect (no cytopathic effect detected after 72 h, cytopathic effect detected after 5 days). ^a^ visible changes to the cells, but no cytopathic effect—individual samples were subcultured (passaged into a new culture vessel); no cytopathic effect was found after passaging. ^b^ visible changes in the cells—detachment of part of the cells from the well bottom.

**Table 4:** Evaluation of FCV viability after exposure to gaseous ozone (CPE+/−: presence/absence of cytopathic effect).

Duration of Exposure	Ozone Generator	Result		
		Site A (CPE+/−; Number of Wells with CPE)	Site B (CPE+/−; Number of Wells with CPE)	Site C (CPE+/−; Number of Wells with CPE)
2 h	A’	+ (3/3)	+ (3/3)	+ (3/3)
4 h	A’	+ (3/3)	+ (3/3)	+ (3/3)
6 h	A’	+ (3/3) ^b^	+ (3/3)	+ (3/3)
4 h	B’	+ (3/3) *	+ (3/3) *	+ (2/3) *
6 h	B’	−	−	−

^b^ visible changes in the cells/detachment of part of the cells from the well bottom. * delayed onset of cytopathic effect visible after 5 days of cultivation.

**Table 5:** Evaluation of FPV viability after exposure to gaseous ozone (CPE+/−: presence/absence of cytopathic effect).

Duration of Exposure	Ozone Generator	Result		
		Site A (CPE+/−; Number of Wells with CPE)	Site B (CPE+/−; Number of Wells with CPE)	Site C (CPE+/−; Number of Wells with CPE)
4 h	A’	+ (3/3)	+ (3/3)	− (0/3) ^a^
6 h	A’	+ (3/3)	+ (3/3)	+ (3/3)
6 h	B’	+ (3/3) *	+ (3/3) *	+ (3/3) ^b,^*

^a^ cytopathic effect found after sample subculturing (passaging into a new culture vessel). ^b^ visible changes in the cells/detachment of part of the cells from the well bottom. * delayed onset of cytopathic effect found after 6 days of cultivation.

## Data Availability

The original contributions presented in the study are included in the article, further inquiries can be directed to the corresponding author/s.
